# What is the Value of Embedding Artificial Emotional Prosody in Human–Computer Interactions? Implications for Theory and Design in Psychological Science

**DOI:** 10.3389/fpsyg.2015.01750

**Published:** 2015-11-12

**Authors:** Rachel L. C. Mitchell, Yi Xu

**Affiliations:** ^1^Centre for Affective Disorders, Institute of Psychiatry Psychology and Neuroscience, King’s College London, London, UK; ^2^Speech Hearing and Phonetic Sciences, Division of Psychology and Language Sciences, University College London, London, UK

**Keywords:** social cognition, emotion, prosody, artificial speech, human–computer interaction

## Abstract

In computerized technology, artificial speech is becoming increasingly important, and is already used in ATMs, online gaming and healthcare contexts. However, today’s artificial speech typically sounds monotonous, a main reason for this being the lack of meaningful prosody. One particularly important function of prosody is to convey different emotions. This is because successful encoding and decoding of emotions is vital for effective social cognition, which is increasingly recognized in human–computer interaction contexts. Current attempts to artificially synthesize emotional prosody are much improved relative to early attempts, but there remains much work to be done due to methodological problems, lack of agreed acoustic correlates, and lack of theoretical grounding. If the addition of synthetic emotional prosody is not of sufficient quality, it may risk alienating users instead of enhancing their experience. So the value of embedding emotion cues in artificial speech may ultimately depend on the quality of the synthetic emotional prosody. However, early evidence on reactions to synthesized non-verbal cues in the facial modality bodes well. Attempts to implement the *recognition* of emotional prosody into artificial applications and interfaces have perhaps been met with greater success, but the ultimate test of synthetic emotional prosody will be to critically compare how people react to synthetic emotional prosody vs. natural emotional prosody, at the behavioral, socio-cognitive and neural levels.

## Introduction

One of the great challenges faced by the human mind is the need to comprehend the mental state of other people. Fortunately, this task is made easier by non-verbal cues in the form of emotional prosody, and it is these such cues that people use to manage their social relationships (Mehu and Scherer, 2012; Tschacher et al., 2014). Prosody refers to acoustic properties beyond those of consonants and vowels, including variables such as pitch, duration, intensity, voice quality, and spectral properties (Ross, 2010). By manipulating prosody, we can alter our tone of voice, and hence change the emotion conveyed. Human–computer interaction concerns how people interact with computerized technology (Boehm-Davis, 2008), and amongst the range of applications and interfaces (HCI-AI) that exist, an ever increasing proportion incorporate artificial speech (Drahota et al., 2008; Robinson and el Kaliouby, 2009). However, a likely obstacle to wide acceptance of today’s artificial speech is its lack of the “human touch,” because it sounds monotonous, and does not change interactively with the user. Whilst an increasing number of HCI-AI now also incorporate rudimentary speech recognition (Putze and Schultz, 2014; Schwenker et al., 2015), they too often lack the “human touch” because they cannot distinguish between the words that are spoken and the way in which they are spoken. In the following mini-review we consider the possible advantages and disadvantages of incorporating expressive speech with emotional prosody into HCI-AI. We examine what has been achieved so far, and the necessary work that remains.

## When is Artificial Emotional Prosody Beneficial for Users?

It has been known for some time that the ability to recognize and express emotions plays a key role in human communication, and more recently, its importance has been recognized in HCI. In research on HCI this is reflected in the wish to achieve more natural interaction (Schröder, 2001), dubbed the “Realism Maximization Theory” (Edlund et al., 2008) Naturally, realism maximization cannot be achieved without incorporating emotional prosody into HCI technology to enhance the communication of intended messages, just as these cues do for natural speech (Esposito et al., 2015). Whilst human and artificial voices can sometimes be distinguished (Belizaire et al., 2007; Gaucher et al., 2013), available evidence suggests that whether synthesized or recorded, a happy voice still makes content seem happier, and a sad voice still makes content seem less happy (Nass et al., 2001).

The key question is, has the aim of enhancing HCI by incorporating synthesized emotional prosody been achieved? Engagement with HCI continues to increase because it is believed that the experience is acceptable and enjoyable, and that HCI-AI can serve as socially intelligent interaction partners that can provide assistance to people (Gorostiza and Salichs, 2011; Wood et al., 2013). But is this really the case? One forum in which HCI-AI appears to be benefitting from incorporating emotional prosody is in the healthcare system. Here, communications to and from patients through emotional channels is of vital importance, and advocates argue that these technologies make HCI-AI more human-like, meaning that users can then rely on well-learned social interaction skills to make the interactions smoother (Berry et al., 2005). Augmentative and alternative communication devices for those unable to produce their own speech (e.g., because of neurodegenerative disease) are a particularly relevant example of the benefits of adding emotional prosody to HCI-AI. As illustrated in the case of the eminent Stephen Hawking, with these devices users can spell out or select any word they choose, but only have recourse to punctuation to influence the way those words are spoken. When listening to users of these devices other than Hawking, listeners may incorrectly assume that they are emotionally as well as speech-impaired, and socially inept (Pullin and Cook, 2013). This is unsatisfactory given the evidence that without the full embodiment of emotional expression present in interpersonal interactions, communication, coordination and performance can suffer immensely (Gerdes et al., 2014). Other applications of expressive speech are covered elsewhere in this review, but for further examples of areas of application which appear promising at this stage, we direct the reader to consider narratives in gaming arenas, voice conversion for the purposes of, and the use of human–human interaction analyses to aid our understanding of social dynamics (Burkhardt and Stegmann, 2009; Schröder, 2009; Vinciarelli et al., 2009; Devillers and Campbell, 2011; Creer et al., 2013; Esposito et al., 2015).

Compared to the expression of emotional prosody, there appears to be greater evidence of success in the incorporation of prosodic emotion recognition into HCI-AI. The driving force here has been the need for these applications to make appropriate reactions in interactive processes, for which the capacity to process human speech signals through emotion recognition is required (Wang et al., 2014; Mavridis, 2015). Whilst many potential applications are being developed (Burkhardt and Campbell, 2014). In the healthcare context, people with autism can sometimes prefer to communicate with computers rather than humans, because it feels more predictable to them and affords greater control over an otherwise chaotic social world (Moore et al., 2000). Sociable HCI-AI incorporating microphones to record emotional prosody may be a good approach to helping social interaction skills (Kim et al., 2013). In this arena, affect sensing and recognition technologies can help increase self-awareness, and provide novel means of self-monitoring (el Kaliouby et al., 2006). Another possible healthcare application is to improve diagnoses of flat affect in patients with depression and schizophrenia, which currently rely on psychiatrists’ subjective judgment (Fragopanagos and Taylor, 2005). Similarly, there is evidence of the promise of automated analyses of vocal markers for Parkinson’s disease (Tsanas et al., 2012). HCI-AI sensitive to emotional prosody also holds promise in the learning environment, e.g., in automatic tutoring applications. Such emotion-sensitive automatic tutors have the capacity to interactively adjust teaching content and the speed at which it is delivered, based on whether a user finds it boring and dreary, or exciting and thrilling (Litman and Forbes-Riley, 2006). Whilst such systems were once feared not to be as effective as one-to-one human tutoring, the addition of the capacity to recognize emotional signals, has narrowed the gap (Mao and Li, 2010).

## When is it not Beneficial?

Unfortunately, the use of artificial speech technology that does not deliver its promise in terms of improved interaction will only frustrate users (Laukka et al., 2011). At the theoretical level, Mori’s “uncanny valley” theory (Edlund et al., 2008) suggests that as artificial HCI characters approach realistic visual similarity to humans, at a certain point they stop being likeable and instead appear eerie, frightening, repulsive—“uncanny” (Mori, 1970). What if the same “uncanny” effect were true for auditory dimensions of HCI-AI, and how might such an auditory effect compromise user acceptance? Certainly disembodied emotional voices presented in isolation are not always received well. Here users may struggle to interpret emotional cues conveyed through the voice, because unlike embodied HCI-AI voices that possess contextual cues to allow users to better determine emotional intentions, disembodied voices allow too much ambiguity (Barker et al., 2013). On a practical level, some question how feasible the development of human-like artificial prosody truly is (Edlund et al., 2008). In particular, there is no singular means of creating artificial prosody, and each specific means has its own imperfections (Esposito and Esposito, 2012). Ultimately, however, the issue boils down to the question of whether and to what extent artificial emotion cues are contextually appropriate, which in turn will be contingent on our level of scientific understanding of emotional expressions.

Questions have also arisen as to the success with which artificial emotional prosody has usefully been incorporated into HCI-AI, in some of the very same areas that heralded its promise. To take the example of ATM’s with emotional prosody, compared to human tellers or traditional ATMs without this capacity, it could be argued that it is a rather unusual experience to talk to a machine (Fischer, 2010). This might be due, in part, to the awkwardness of knowing that no human is there, and it might also be difficult to imagine that for ordinary use, people want to have an artificial agent openly express anger or displeasure to a user. Or it might simply reflect that this technology is relatively new, and that consumers and users need time to get used to such well-intentioned amplification of emotional communication through prosody. A second exemplar concern is the healthcare applications for those unable to produce their own speech. Here there is no vocal individuality, i.e., the systems are not designed to imitate a specific speaker’s voice (Wendemuth and Biundo, 2011). This identity mismatch may impact use and adoption of these devices and further perpetuate the divide between the user and the device (Mills et al., 2014).

## What about the Future?

If it were possible to further improve the quality of artificial speech with emotional prosody, it would have significant consequences for those involved in creating HCI-AI (Brenton et al., 2005). As computerized technology becomes an ever greater fixture at home and at work, our future interactions with it will need to become even more sophisticated (Wendemuth and Biundo, 2011; Honold et al., 2014). Some time ago, it was recommended that artificial speech synthesis technology should not only have the ability to control prosody based on meaning, but also the capability to control individual speaking style (another form of prosody), choosing application-oriented speaking styles, and be able to add emotion (Furui, 1995). Yet, as we have seen, there remains much work to be done (Burkhardt and Stegmann, 2009). Although problematic, delivery to date of HCI-AI able to crudely interact with people, attempt to sense emotional prosody, and try to produce suitable responses, has produced great expectations for the future (Esposito and Esposito, 2012).

Social HCI-AI need to follow behaviors similar to humans: they interact and communicate with humans by following a set of social rules (Pullin and Cook, 2013). Additional work on the social skills and responsivity with which HCI-AI are programmed will likely increase the empathy and acceptance level of interactions further (Leite et al., 2013). From the human interface point of view, it has long been recognized that HCI-AI should be able to automatically acquire new knowledge about the thinking process of individual users, automatically correct user errors, and understand user intentions by accepting rough instructions and inferring details (Furui, 1995). Ultimately, the hope for the future is that HCI-AI could extract the prosodic cues from a user’s speech, capitalize on the information to inform predictive models of likely emotions (Litman and Forbes-Riley, 2006), and amend their own displays and actions accordingly. Such an aim is not without its challenges though. For example, much work is ongoing at present into how a HCI-AI might best transcribe and annotate a user’s prosodic emotion cues in order to reliably label and act appropriately on the likely emotional state conveyed thus (Siegert et al., 2013, 2014). Beyond being responsive and interactive, HCI-AI with emotional prosody also requires further work on the modification of their implementation depending on context. We may adopt a different palette of tones of voice with different people, depending on our relationship to them or the social context (Pullin and Cook, 2013).

Whilst there may be problems with current technology (Schröder, 2009), we believe that healthcare applications of HCI-AI with artificial emotional prosody still hold the potential to make a genuine difference to peoples’ lives in the future. HCI-AI that express emotion cues could especially enhance the ability to make sense of and communicate with others in people with difficulty understanding, communicating and regulating their emotion systems, such as autism, and the affective disorders (Robinson and el Kaliouby, 2009). Personalized voices may also be possible for those who rely on alternative or augmented communication devices, by mapping existing text to speech corpora onto a voice personalized to the residual vocal characteristics of a specific user (Creer et al., 2013; Mills et al., 2014). Whilst future assistive HCI-AI agents with emotional prosody might be expected to benefit users of all ages, the development of socially intelligent assistive technology has promise for increasing quality of life for older adults in particular, in the form of reminder systems, telecommunication systems, surveillance systems, and the ability to provide social interaction and complete daily household tasks (Beer et al., 2015).

## Problems Still Requiring a Solution

As alluded to above, current technology for synthesizing effective emotional prosody is still rudimentary. Yet if we were able to improve its quality, it would have far-reaching consequences for the success of HCI-AI attempting to capitalize on its potential (Brenton et al., 2005). One source of the slow progress toward synthesizing good quality artificial emotional prosody is the difficulty of identifying clear acoustic correlates for discrete emotions (Banse and Scherer, 1996; Schröder, 2009). This problem is compounded by the fact that most work on natural emotional prosody has taken its measurements from recordings of actors trying to portray various emotional tones of voice. But it is questionable whether actors’ portrayals authentically represent the characteristics of speech used by ordinary people when they spontaneously experience emotions (Douglas-Cowie et al., 2007; Esposito and Esposito, 2012). A further difficulty is that, because each acoustic dimension can be measured in many different ways (Xu, 2011), there are actually many more possible acoustic measurements than can be realistically exhausted in elucidating acoustic correlates. However, recent work on defining emotions through mathematical modeling holds promise in tackling the current lack of consistent acoustic correlates in human communications (Hartmann et al., 2012; Xu, 2015).

A second problem with generating good quality artificial emotional prosody is that there is still an unsolved need to directly control specific aspects of artificial prosodic emotion cues based on theoretical motivations, as attempted in dimensional approaches to the measurement of emotions (Burkhardt and Stegmann, 2009; Mauss and Robinson, 2009; Verma and Tiwary, 2014). However, effective control methods for empirically investigating vocal emotional expressions have not yet been developed, and although some links have been found between activation (arousal) and parameters such as pitch and intensity, F_0_ range, articulation rate etc., no acoustic parameters have been identified as reliable indicators of key dimensions of emotion such as valence or approach/avoidance (Mauss and Robinson, 2009). To address the lack of theoretical foundation, robust algorithmic implementation of situated social information processing facets is necessary, and it is likely that multiple theoretical perspectives will need to be considered, ranging from mathematical models and dynamics of signal exchanges (e.g., emotional states and context effects), to social intelligence, behavioral analyses, and cognitive processes such as cooperation (Esposito et al., 2015).

An interesting recent development is an ethological approach to emotional prosody, which examines commonalities been animal calls and human emotional prosody (Xu et al., 2013a). The theoretical framework was first developed in a study of animal calls (Morton, 1977) and later extended to humans (Ohala, 1984). It posits a strong selection pressure for organisms to vocally (just as they do visually) manipulate their apparent body size when interacting with others. This size-projection hypothesis has been shown to be capable of explaining a broad range of animal and human behaviors, as well as bodily anatomies, including the acoustic characteristics of animal calls (Fitch and Kelley, 2000; Reby and McComb, 2003; Reby et al., 2005; Harris et al., 2006; Charlton et al., 2007, 2011), the descent or elongation of the vocal tract (Fitch, 1999; Fitch and Reby, 2001), and sexually and socially related human vocal behavior (Feinberg et al., 2005, 2006, 2008; Bruckert et al., 2006; Riding et al., 2006; Fraccaro et al., 2011; Xu et al., 2013b). The relevance of this hypothesis for human emotional prosody has been demonstrated by a series of studies (Chuenwattanapranithi et al., 2008; Noble and Xu, 2011; Xu et al., 2013a,b). The consistency of results shown in these studies suggest that the ethological-based approach is promising and needs to be further explored in future research.

## Evaluation of Success

In this review, we have seen that the ability to synthesize emotional prosody might, for the most part, be desirable (Drahota et al., 2008; Robinson and el Kaliouby, 2009). As work continues, it will be important to understand and properly evaluate our predispositions to artificial emotional prosody (Robinson and el Kaliouby, 2009). It is particularly vital to know whether these cues are perceived in the same way as human emotion cues. After all, the most critical test is to put artificial speech technology into action and to expose it to the critical comparison with social reality as created by nature (Vogeley and Bente, 2010). To make such a judgement, we believe that a wide array of assessments will be required.

At the behavioral level, some literature suggests people may not be as good at identifying synthesized facial expressions in avatars as they are at identifying human expressions (Moser et al., 2007; Rosset et al., 2008), perhaps because many are clearly not perceptually natural (Douglas-Cowie et al., 2007; Cowie, 2009). However, other studies have shown that recognition of synthesized facial expressions can sometimes match or even surpass that of human expressions (Dyck et al., 2008). Whilst human and artificial voices can be distinguished at the behavioral level (Nass et al., 2001; Belizaire et al., 2007), a clear main effect has not been demonstrated when judging intonational emotions. Another complication comes from the fact that facial studies report that identification of emotion from artificial faces may be emotion-dependent, and that recognition is worst for disgust (Moser et al., 2007; Dyck et al., 2008). The uncanny valley theory might more generally predict that negative valence emotions (aversive or warning stimuli) such as anger, fear, sadness and disgust might attract lower ratings of familiarity and human-likeness (Tinwell et al., 2011). The validity of such predictions has never been tested for artificial emotional prosody.

Of particular relevance at the socio-cognitive level, would be to determine what criteria afford a machine the status of “social agent” (a software agent or robot capable of social communication with human beings; Aharoni and Fridlund, 2007). Despite the knowledge that computerized technology does not warrant social treatment, people nonetheless tend to apply social expectations to and exhibit the same responses to computerized technology as they would to human communication partners (Lee, 2010). Indeed, there is prior evidence for impression formation from artificial emotional prosody. For example, its implementation has been shown to influence social judgments of liking and credibility from synthesized speech (Nass et al., 2001), and people tend to present themselves in a more positive light to HCI agents that emit artificial speech (Parise et al., 1999), although findings of impression management in response to artificial speech are not as strong as that in response to human speech (Lee, 2010; Mitchell et al., 2011). We suggest that the social influence of HCI-AI agents with artificial emotional prosody may even extend to stereotypical impression formation. Indeed, the literature on “Sensitive Artificial Listeners” illustrates that it is possible for two people to have a conversation in which one pays little or no attention to the meaning of what the other says, and chooses emotional interpretations and subsequent responses on the basis of quite superficial cues(Douglas-Cowie et al., 2008). Recent data has even shown that participants might prefer robots with matching gender-occupational role stereotypes (Tay et al., 2014), and it would be easy enough to test the hypothesis that emotional prosody makes synthesized speech more human-like, and therefore more susceptible to human-like stereotypes.

In assessing the utility of artificial emotional prosody at the neural level, the activation of “social cognition” brain regions is more significant for human stimuli than for artificial stimuli, as if our brains are “tuned in” to the former (Vogeley and Bente, 2010). Most pertinent, this is observed when participants derive emotion cues from facial expressions, in the amygdala, insula and prefrontal cortex (Mullennix et al., 2003; Moser et al., 2007; Cheetham et al., 2011; de Borst and de Gelder, 2015). Thus it could be possible to use the activation intensity of such brain regions to assess how human-like an artificial emotion stimulus is (Chaminade and Cheng, 2009). With respect to prosody, researchers should also measure the activation of regions associated with “voiceness” (degree to which an auditory stimulus resembles the human voice) i.e., bilateral upper banks of the superior temporal sulcus (Belizaire et al., 2007), and those associated with prosodic emotion recognition, i.e., the right posterior middle/superior temporal gyri (Mitchell et al., 2003). We would recommend that the neural response of emotion-specific regions such as the amygdala for fear (Janak and Tye, 2015), insula for disgust (Chapman and Anderson, 2012), and superior temporal sulcus for anger (Carter and Pelphrey, 2008) should also be probed in evaluating artificial emotional prosody. However, it needs to be borne in mind that there is no singular means of creating artificial prosody. Thus each specific means of synthesizing emotional prosody will have its own imperfections, its own acoustic correlates, and might invoke its own pattern of neural response.

## Concluding Thoughts

Given the importance of emotional prosody in human to human communication, there is significant potential for interactions between humans and computerized technology to benefit from including synthesized emotional prosody in HCI-AI. This need is only amplified by the pace with which HCI-AI are evolving. Indeed, to quote Picard “If we want computers to be genuinely intelligent and to interact naturally with us, we must give computers the ability to recognize, understand, and even to have and express emotions” (Picard, 1997). Whilst HCI has mostly been enhanced by including artificially synthesized *facial* expressions, a full multi-level evaluation of our reactions to synthesized emotional prosody is needed before the wisdom of its inclusion can be properly evaluated. Further work is also necessary to determine whether there are circumstances in which its inclusion does not work well, or whether the problem lies in how it is synthesized. Achieving human-likeness dialogs with HCI-AI through explicit computational models might also provide valuable insights about how humans communicate with each other (Edlund et al., 2008).

### Conflict of Interest Statement

The authors declare that the research was conducted in the absence of any commercial or financial relationships that could be construed as a potential conflict of interest.
